# Mucosal Immunity against SARS-CoV-2 in the Respiratory Tract

**DOI:** 10.3390/pathogens13020113

**Published:** 2024-01-26

**Authors:** Hae-Eun Noh, Min-Seok Rha

**Affiliations:** 1 Department of Otorhinolaryngology, Yonsei University College of Medicine, Seoul 03722, Republic of Korea; haun1812@gmail.com; 2 Department of Biomedical Sciences, Yonsei University College of Medicine, Seoul 03722, Republic of Korea

**Keywords:** SARS-CoV-2, COVID-19, respiratory tract, airway, mucosal immune response, allergic airway

## Abstract

The respiratory tract, the first-line defense, is constantly exposed to inhaled allergens, pollutants, and pathogens such as respiratory viruses. Emerging evidence has demonstrated that the coordination of innate and adaptive immune responses in the respiratory tract plays a crucial role in the protection against invading respiratory pathogens. Therefore, a better understanding of mucosal immunity in the airways is critical for the development of novel therapeutics and next-generation vaccines against severe acute respiratory syndrome coronavirus 2 (SARS-CoV-2) and other respiratory viruses. Since the coronavirus disease 2019 pandemic, our knowledge of mucosal immune responses in the airways has expanded. In this review, we describe the latest knowledge regarding the key components of the mucosal immune system in the respiratory tract. In addition, we summarize the host immune responses in the upper and lower airways following SARS-CoV-2 infection and vaccination, and discuss the impact of allergic airway inflammation on mucosal immune responses against SARS-CoV-2.

## 1. Introduction

Since the emergence of the initial case of pneumonia with an unknown cause in Wuhan, China, in December 2019 [1], the novel severe acute respiratory syndrome coronavirus 2 (SARS-CoV-2) has rapidly spread worldwide. SARS-CoV-2 infection causes coronavirus disease 2019 (COVID-19), which manifests a broad spectrum of clinical presentations, ranging from asymptomatic infection to severe disease [2]. COVID-19 has caused global morbidity and devastating disruptions in daily life. Prophylactic vaccines using various platforms were developed during the COVID-19 pandemic, and their administration started in populations worldwide in December 2020. Despite improved protection against SARS-CoV-2 infection through the development of vaccines, a deeper understanding of the immune response to SARS-CoV-2 will help guide the development of next-generation vaccines or therapeutics against respiratory viral infections. This knowledge will contribute to improved preparedness for future pandemics.

The COVID-19 outbreak and global efforts to overcome the pandemic have substantially increased our knowledge of immune responses against respiratory viruses, particularly in the human system. However, although blood cells are not the primary site of SARS-CoV-2 infection, the majority of the analysis of the immune response has been limited to peripheral blood (PB). The nasal cavity is the site of both viral entry and the initial replication of SARS-CoV-2 [3]. SARS-CoV-2 spreads to the epithelial cells of the lower respiratory tract and causes pneumonia, particularly in cases of severe COVID-19 [4], whereas limited levels of viral replication can occur in other tissues [5,6]. Respiratory viruses, such as the coronavirus and influenza virus, are potential causes of future pandemics and primarily infect the respiratory mucosa; therefore, it is important to understand and measure local immune responses in the airway.

Several reviews have discussed the various aspects of the systemic immune response to SARS-CoV-2 following infection and vaccination [7,8]. In the current review, we focus on the local immune response to SARS-CoV-2 in the airway. We summarize the current knowledge of the characteristics of the mucosal immune system in the respiratory tract, and discuss airway immune responses in the context of COVID-19. Furthermore, we describe alterations in mucosal immune responses against SARS-CoV-2 under allergic airway inflammation conditions.

## 2. An Overview of the Characteristics of Respiratory Mucosal Immunity

### 2.1. Epithelial Barrier

The epithelial barrier serves as the first line of defense against invasion by respiratory pathogens and allergens in the airways. It includes cell–cell junctions that function as a physical barrier, mucociliary clearance for mechanically removing inhaled pathogens and allergens the commensal microbiota, immunoglobulins (Igs), and defense molecules [9]. These are crucial for trapping and cleansing risk factors and modulating the immune response. Cell–cell junctions comprise various structures, including tight junctions (TJs), adherens junctions (AJs), gap junctions, desmosomes, and hemidesmosomes. In particular, apical junction complexes (AJCs) play an important role in epithelial barrier function. The AJC consists of the most apically located TJs and underlying AJs [10]. TJs regulate the movement of ions and molecules, as well as paracellular transport, whereas AJs are essential for initiating and maintaining cell–cell adhesion [11]. TJs are composed of proteins, such as occludin (OCLN), claudin, junctional adhesion molecules, and zonula occludens (ZO), whereas AJs contain complexes such as cadherin/catenin and nectin/afadin [12]. The expression of these genes and proteins in the AJC serves as an indicator of epithelial barrier function and transepithelial electrical resistance (TER) [13]. In addition, commensal microbiota plays a defensive role against viral infections in the upper respiratory tract by modulating-type I and type III interferon (IFN)-mediated immune mechanisms [14,15,16]. Nasal commensal *Staphylococcus epidermidis* was reported to provide initial antiviral protection against influenza A virus infection by enhancing the IFN-λ-dependent innate immune response in the nasal mucosa [16].

Epithelial barrier dysfunction can be caused by several factors, including mucociliary dysfunction and microbial dysbiosis. In particular, the disruption of epithelial barrier integrity leads to a decrease in TER and increased barrier permeability. This disruption can occur owing to various pathogens, allergens, or other pathological conditions. The infection of human rhinovirus in human nasal epithelial cells in vitro leads to a decreased expression of membrane proteins, including ZO-1, E-cadherin, claudin-1, and OCLN [17,18]. Similar alterations were observed after the infection of bronchial epithelial cells (BECs), where a reduced expression of TJ proteins at the epithelial barrier, decreased TER, and the dissociation of ZO-1 from the TJs was observed [19]. Several studies have reported defective epithelial barriers in patients with allergic rhinitis (AR), chronic rhinosinusitis, and asthma [20,21,22]. The expression of CLND-1, OCLN, and ZO-1 proteins was found to be significantly reduced in the BECs of asthmatic children compared to that in non-asthmatic controls [19]. House dust mites (HDMs) are an AR allergen and exhibit proteolytic activity that cleaves TJ proteins [23,24]. Steelant et al. reported a decrease in OCLN and ZO-1 expression along with increased epithelial permeability in the nasal epithelium of patients with HDM-induced AR [22].

### 2.2. Innate Immunity

Airway surface fluid is composed of mucus, antimicrobial peptides (AMPs), and enzymes, playing a crucial role as the first line of host defense in nature. Mucus is produced by mucous glands and goblet cells, capturing inhaled debris and pathogens and facilitating mucociliary clearance through ciliary beating. The primary components of airway mucus are O-glycosylated mucin glycoproteins, categorized as gel-forming and transmembrane mucins. In gel-forming mucins, MUC5AC and MUC5B play a crucial role in effectively clearing pathogens through mucociliary clearance. Among the transmembrane mucins, MUC1, MUC4, and MUC16 are the major ones, with MUC1 being the most abundant. While MUC1 and MUC4 are present in both the upper and lower respiratory tracts, MUC16 is exclusively expressed in the lower tract. These mucins create a barrier in the respiratory epithelium, acting as decoy receptors against pathogens. Additionally, they contribute to mucociliary clearance by shedding the extracellular domain, which binds to pathogens and releases them into the lumen, from the cell surface [25]. AMPs include cationic defensins like human β-defensins (HBDs) 1, 2, 3, 4, human cathelicidins (LL-37), and secretory leukocyte protease inhibitor (SLPI). They exhibit synergistic activity with host defense molecules like lysozymes and lactoferrin. AMPs are upregulated during infections, demonstrating microbiocidal effects and immune modulation activities such as chemotactically attracting immune cells and modulating cytokine production [26].

The immune response to viral infections is initiated by the innate immune system, which recognizes pathogens and induces the production of pro-inflammatory cytokines and chemokines. Innate responses are triggered when immune cells with pattern recognition receptors (PRRs) recognize pathogen-associated molecular patterns (PAMPs) which include viral molecules, such as viral RNA or oxidized phospholipids, or damage-associated molecular patterns (DAMPs), such as endogenous host molecules released from damaged and dying cells. PRRs, such as Toll-like receptors (TLRs), retinoic acid-inducible gene (RIG-I)-like receptors (RLRs), nucleotide-binding oligomerization domain (NOD)-like receptors (NLRs), and other cytosolic virus sensors, initiate the immune response to viral infections [27].

After the recognition of PAMPs and DAMPs, antiviral responses are activated through type I or III interferon (IFN) signaling pathways and the production of pro-inflammatory cytokines and chemokines, ultimately suppressing viral replication, eliminating infected cells, and initiating an adaptive immune response [28,29]. Viral recognition leads to IFN induction via three major pathways: cyclic guanosine monophosphate-adenosine monophosphate synthase/stimulator of IFN genes (cGAS/STING), TLR/TIR-domain-containing adapter-inducing IFN-β (TRIF)/Myeloid differentiation factor 88 (MyD88), and RLR/mammalian mitochondrial antiviral signaling protein (MAVS) pathways. All pathways activate the kinase TBK1 which phosphorylates the transcription factors IFN regulatory factor (IRF)3 and IRF7, thereby stimulating IFN production. They also activate the nuclear factor-κB (NF-κB) family of transcription factors and the subsequent production of pro-inflammatory cytokines [30].

Type I IFNs (IFN-α, IFN-β) bind to IFNAR (IFN-α/β receptor), and type III IFNs (IFN-λ 1,2,3,4) bind to IFNLR (IFN-λ receptor). Type I and III IFNs are key players in the antiviral innate immune response and activate several signaling cascades, including the JAK/STAT pathway. Subsequently, they form IFN-stimulated gene (ISG) factor 3 (ISGF3) complexes, driving the expression of hundreds of ISGs that primarily elicit an effective antiviral response [28,31,32,33]. IFN-λ acts in a more targeted fashion, as the expression of its receptor IFNLR1 is limited to mucosal epithelial cells of the respiratory, gastrointestinal, and reproductive tracts and specific immune cells, whereas IFNAR is expressed in most cells throughout the body [34,35]. Respiratory epithelial cells respond to both IFN-λ and IFN-α/β. In general, during the early stage of infection, an immune response based on IFN-β and IFN-λ is produced by infected cells, which is probably local. However, as the infection progresses, plasmacytoid dendritic cells become major cellular sources of IFN-α, leading to a more systemic response [36]. In some respiratory virus infections, respiratory epithelial cells primarily produce IFN-λ rather than IFN-α and IFN-β, implying a key role of IFN-λ in mediating antiviral immunity in the respiratory tract [37,38]. These results suggest a significant potential for type III IFN to be used as a therapeutic target for respiratory viral infections, considering its higher tissue specificity compared to type I IFN.

In addition, dendritic cells (DCs), as one of the most potent types of antigen-presenting cells, play a crucial role in innate immunity and contribute significantly to antiviral responses. They consist of two main functional subtypes: conventional or myeloid DCs (cDCs) and plasmacytoid DCs (pDCs). cDCs include CD1c^+^, CD16^+^, and CD141^+^ cDC subsets and also monocyte-derived DCs generated by circulating monocytes in the presence of inflammation [39]. In human tissues, CD141-expressing cDC1s in the vascular wall and mucosa stimulate Th1 responses, while CD1c-expressing cDC2s in the lamina propria produce inflammatory chemokines, facilitating immune cell aggregation, and may contribute to immune tolerance [40]. pDCs are present throughout the lung tissue including the airway. They play a vital role in innate immunity by producing a substantial amount of type I IFN during early antiviral responses through the stimulation of TLR 7/9 [39].

### 2.3. Tissue-Resident Memory T Cells in the Respiratory Tract

Following the innate immune response, the adaptive immune system is activated to control and eradicate infections. Despite the importance of humoral immune responses, coordinated cellular immunity is also essential for disease control [41,42]. Sterilizing immunity is primarily mediated by antibodies; however, insufficient antibody levels or mutations in antibody-binding sites may cause reinfection. In such scenarios, memory T cell responses are crucial for the prevention of viral dissemination and progression into severe disease, particularly in the context of infections with variants. Previous studies have shown that virus-specific memory T cells are long-lasting after recovery from viral infection [43,44]. Robust T-cell responses in the respiratory tract may play a crucial role in impeding disease progression by rapidly exerting effector functions, given that re-exposure to respiratory viruses primarily occurs in the airway [45].

Tissue-resident memory T (T_RM_) cells are a subset of memory T cells characterized by long-term residency in non-lymphoid tissues [46,47]. T_RM_ cells are found in almost all peripheral tissues, including the skin [48], lung [49,50], gut [51], brain [52,53], and genital tract [54]. T_RM_ cells from various tissues exhibit a common gene expression profile, in addition to some tissue-specific differences in gene expression. In animal models, the tissue-residency of T cells can be determined by various experimental methods, including parabiotic surgery, in vivo antibody labeling, T cell depletion, and tissue transplantation [47]. While these techniques rigorously determine T_RM_ cells, their application in humans has clear limitations. Thus, phenotypic or transcriptional profiling is the primary method for the identification of T_RM_ cells in humans. T_RM_ cells are frequently defined by the expression of surface markers associated with tissue retention [46,47]. As CD69 promotes tissue retention by downregulating sphingosine-1-phosphate receptor-1 (S1PR1) that is required for tissue egress [55], CD69 is the canonical marker of T_RM_ cells. In addition to CD69, other surface markers, including adhesion molecules and tissue-homing chemokine receptors, characterize T_RM_ phenotypes. However, the expression of these surface markers on T_RM_ cells varies across tissues. CD103 (αE integrin), which binds to E-cadherin on epithelial cells, is highly expressed on T_RM_ cells in epithelial tissues such as the skin, intestines, and lungs [47]. In contrast, most CD8^+^ T_RM_ cells in the liver and secondary lymphoid organs lack CD103 expression [56,57,58]. Several studies have reported that CD103^+^CD4^+^ T_RM_ cells are also present in barrier tissues [59,60]. Collectively, the combination of CD69 and CD103 may serve as a reliable marker for identifying human T_RM_ cells, especially in epithelial tissues. CD49a (integrin α1), which forms very late antigen-1 with CD29 (integrin β1), is expressed on a subset of CD4^+^ and CD8^+^ T_RM_ cells in diverse tissues [57,60,61,62,63]. A recent study in mice demonstrated that CD49a facilitates the locomotion of virus-specific CD8^+^ T_RM_ cells within the lungs [64]. In addition, the tissue retention of CD8^+^ T_RM_ cells is impaired in the absence of very late antigen-1 [62]. Furthermore, T_RM_ cells express a variety of tissue-homing chemokine receptors required for localization in tissues. CXCR6, the chemokine receptor for CXCL16, is highly expressed in human T_RM_ cells in the lungs [65], liver [57], and lymphoid tissues [60]. Studies in mice have demonstrated that CXCR6 is required for the recruitment of CD8^+^ T_RM_ cells to the airway epithelium [65]. CXCR3, the receptor for the chemokines CXCL9, CXCL10, and CXCL11, is also expressed in a proportion of lung T_RM_ cells [49,66].

T_RM_ cells rapidly exert effector functions upon a secondary pathogen encounter and restrict disease progression. Accumulating evidence has shown a protective role for airway T_RM_ cells against respiratory viral infections. An experimental respiratory syncytial virus (RSV) challenge in healthy adult volunteers showed that the abundance of pre-existing virus-specific CD8^+^ T_RM_ cells in bronchoalveolar lavage fluid (BALF) before infection correlated with reduced symptoms and viral loads [67]. In a mouse model, the transfer of airway T_RM_ cells from previously infected animals led to the protection against RSV infection [68]. In the mouse model of coronavirus infections, protection from disease was primarily mediated by virus-specific CD4^+^ T_RM_ cells in the upper airway to promote the secretion of IFN-γ and the recruitment of virus-specific CD8^+^ T cells [69]. Several studies have also demonstrated that lung CD4^+^ and CD8^+^ T_RM_ cells confer protective immunity against influenza virus infection [66,70]. Virus-specific T_RM_ cells are also critically required for optimal protection from infection with heterosubtypic influenza viruses [71]. Furthermore, a seminal study has shown that nasal CD8^+^ T_RM_ cells limit viral spread to the lungs and reduce the severity of pulmonary diseases [72].

### 2.4. Tissue-Resident Memory B Cells and Antibody-Secreting Cells in the Respiratory Tract

Similar to memory T cells, memory B cells persist long-term and rapidly differentiate into antibody-secreting cells upon antigen re-encounter [73]. Memory B cells can re-enter the germinal center upon antigen re-exposure and undergo further affinity maturation [74]. Antibodies produced by plasma cells that are terminally differentiated cells contribute to the defense against pathogen reinfection [75]. The cooperation between memory B cells and plasma cells confers robust protection against pathogens [74]. The proximity of antigen-presenting cells, memory follicular helper T (T_FH_) cells, and memory B cells facilitates robust recall responses.

Analogous to T_RM_ cells, recent data have revealed the existence of a specific subset of memory B cells residing in peripheral tissues, known as tissue-resident memory B (B_RM_) cells [76,77,78,79,80]. Allie et al. conducted a parabiosis surgery and intravenous antibody labeling and demonstrated that pulmonary influenza infection elicited lung B_RM_ cells with distinct phenotypes compared to their systemic counterparts [76]. Similarly, another study showed that intranasal immunization induced IgA-producing B_RM_ cells in the lungs [77]. Specific markers for the identification of B_RM_ cells are required to investigate their characteristics. To identify specific markers for B_RM_ cells, the transcriptional profiles of both murine and human B_RM_ cells have been analyzed [79,81]. The results showed that lung memory B cells exhibited a higher expression of CXCR3 and CD69 than their counterparts in the mediastinal lymph nodes and spleen. The downregulation of *CCR7*, *SELL*, *S1PR1*, and *KLF2*, and the upregulation of *CXCR3*, *CCR6*, and *CD69* were observed in lung B_RM_ cells [79]. Consistent with these results, the upregulation of CD69, CXCR3, and CCR6, the chemokine receptor for CCL20, has been observed in memory B cells of the human lungs [78,79]. Most CD27^+^ memory B cells from the human gut also express CD69 [80]. These results suggest that B_RM_ cells share common signatures underlying tissue residency with T_RM_ cells in non-lymphoid organs. However, the transcriptional program determining the differentiation and fate of memory B cells remains unclear.

Humoral responses to pathogen re-encounters have been investigated in peripheral tissues. B_RM_ cells rapidly differentiate into antibody-secreting cells during secondary infections and subsequently secrete antibodies against pathogens [76]. In influenza infections, reinfection induces the formation of inducible bronchus-associated lymphoid tissue, which supports the maturation and selection of B cells, thereby generating B_RM_ cells as well as resident memory T_FH_ cells [82]. In addition, mice with IgA-producing lung B_RM_ cells following local immunization show superior protection against secondary challenges with both homologous and heterologous strains of the influenza virus, supporting the cross-reactivity of local humoral immunity [77]. Intriguingly, both CXCR3^+^CCR6^+^ virus-specific B cells and CXCR3^−^CCR6^+^ bystander B cells are generated in the lung after infections with the influenza virus and SARS-CoV-2 [83]. However, the potential benefits of bystander B cells remains to be elucidated. In the case of influenza reinfection, alveolar macrophages are key initiators of humoral recall responses through the secretion of IFN-γ and CXCR3 ligands, which in turn activate the recruitment of CXCR3^+^ B_RM_ cells to the infection site [84]. Although it remains unclear whether memory T_FH_ cells are critically required for the initiation of these recall responses, it can be assumed that B_RM_ cells as well as resident memory T_FH_ cells are recruited close to alveolar macrophages, thereby exerting robust protective immunity.

## 3. SARS-CoV-2 Entry into the Respiratory Tract

The coronavirus virion contains spike (S), envelope (E), and membrane (M) proteins, with an RNA genome complexed with nucleocapsid (N) proteins to form a helical capsid [85]. The S protein is anchored to the viral envelope and is composed of S1 and S2 subunits. During infection, two cleavage events occur for cellular entry: the cleavage at the junction of the S1 and S2 subunits and at the S2′ site, located upstream from the fusion peptide within the S2 subunit [86]. In SARS-CoV-2, the S1–S2 boundary is cleaved by furin during virus maturation [87], and when the S protein binds to the angiotensin-converting enzyme 2 (ACE2) entry receptor of the host target cell, the S2′ cleavage site is exposed [88]. The exposed S2′ site is cleaved by different host proteases depending on the entry route. If the transmembrane protease serine subtype 2 (TMPRSS2) is sufficiently expressed near ACE2 on the target cell, the virus-ACE2 complex encounters TMPRSS2 at the cell surface, leading to S2′ cleavage followed by membrane fusion to release viral RNA into the cell cytoplasm [89,90]. This step must occur after ACE2 binding to ensure viral S protein activation [91,92]. The S protein cleavage by TMPRSS2 facilitates early entry into the cell membrane as opposed to late entry through the endosome. Although TMPRSS2 can be replaced by other proteases, its binding to the ACE2 receptor is essential for cell entry. If TMPRSS2 expression is insufficient and the complex does not encounter TMPRSS2, the virus-ACE2 complex is internalized into endosomes. Cleavage at the S2′ site is then performed by low pH-triggered cathepsin in the endosomes, leading to the fusion with the endosomal membrane and the release of viral RNA into the cell cytoplasm [88,93].

Following cellular entry, genomic RNA is translated into two large polyproteins, pp1a, corresponding to NSP1 to NSP11 from open reading frame (ORF)1a, and pp1ab, corresponding to NSP12 to NSP16 from ORF1b. These encode 16 nonstructural proteins (NSPs) that facilitate the formation of the viral replication–transcription complex [94]. The SARS-CoV-2 genome encodes structural proteins, including S, E, M, and N, as well as accessory proteins, including ORF3a, 3b, 6, 7a, 7b, 8, 9b, 9c, and 10. These ORFs and NSPs play crucial roles in viral replication and the evasion of the host immune response [95]. Replicated genomic RNA and structural proteins are assembled in the endoplasmic reticulum–Golgi intermediate compartment and the fully formed virions are exocytosed [96].

Numerous studies have focused on the expression levels of ACE2 rather than TMPRSS2 to understand the differences in SARS-CoV-2 infection risk and clinical outcomes [97,98]. The co-expression of ACE2 and TMPRSS2 within cells is considered important for the entry of SARS-CoV-2. Notably, these viral entry-associated genes of SARS-CoV-2 are known to be highly expressed in the nasal epithelial cells [3,99], and their expression in the upper respiratory epithelium is believed to have a positive correlation with viral susceptibility and transmissibility. An average three-fold increase in ACE2 expression has been observed in secretory cells from patients with COVID-19 compared to control individuals [4]. Notably, SARS-CoV-2 causes a decrease in epithelial barrier function and the disruption of TJs. It also perturbs ciliogenesis and downregulates Foxj1 expression, leading to the loss of ciliated epithelial cells and the impairment of mucociliary clearance [100]. These results suggest a pathogenic mechanism that underlies SARS-CoV-2 spread in the respiratory tract.

## 4. Innate Immunity to SARS-CoV-2 Infection in the Airway

Innate immune defense plays a key role in controlling SARS-CoV-2 infections, and deficiencies in the innate immune system can trigger severe COVID-19 [101,102]. Zhang et al. reported that patients with inborn defects in TLR3- and IRF7-dependent type I IFN immunity are prone to life-threatening COVID-19 pneumonia [102]. Bastard et al. reported that the presence of autoantibodies against type I IFN-α2 and IFN-ω is associated with a high risk of severe COVID-19 [101].

Accumulating evidence supports that both soluble and transmembrane mucins play important roles in SARS-CoV-2 infection, but whether they are protective or pathogenic still remains controversial. Mucus hypersecretion may exhibit a negative impact on disease development or progression due to reduced mucocililary clearance. Indeed, the protein levels of MUC1 and MUC5AC were elevated in airway mucus of patients with COVID-19 compared to control individuals [103], and a high production of MUC5AC was observed in SARS-CoV-2-infected primary respiratory [104]. Other researchers also showed that SARS-CoV-2 infection is associated with a high prevalence of MUC5B-dominated mucus plugging in the distal lung, and MUC5B expression was increased in airway regions of COVID-19 autopsy lungs [105]. These data suggest that mucolytic agents may be therapeutics for COVID-19. In contrast, a recent study reported in vitro evidence of protective functions of the glycosylated extracellular domains of transmembrane mucins in different respiratory cell types by preventing SARS-CoV-2 binding and entry [106]. A recent study conducting genome-wide CRISPR screens showed that the overexpression of transmembrane mucins MUC1, MUC4, or MUC21 reduced SARS-CoV-2 infection compared to cells with a non-targeting guide [107]. Further in vivo studies need to address the precise role of each mucin in the pathophysiology of COVID-19.

AMPs, such as defensins, may also play a role in protection against SARS-CoV-2 infection. A previous study showed that β-defensin transcripts were increased in the nasopharyngeal swab samples from patients with SARS-CoV-2 infection compared to those from control individuals [108]. Recent research also suggests that human defensins may inhibit SARS-CoV-2 infection by blocking viral entry [109]. The precise role of AMPs needs to be further elucidated.

Similar to other respiratory viruses, SARS-CoV-2 RNAs are mainly recognized by TLRs and RLRs, including RIG-I and MDA5 [110,111,112]. TLRs activate the TRIF/MyD88 signaling pathway, and RLRs (RIG-I and MDA5) activate the MAVS pathway, leading to the production of various cytokines, including pro-inflammatory cytokines such as TNF-α, IL-1β, IL-6, and type I and III IFNs [113]. NLRs, such as NLRP3 inflammasome, and the cGAS–STING signaling pathway, which is activated upon the detection of cytosolic DNA from damaged host mitochondria, have also been reported to sense SARS-CoV-2 infection and induce the production of type I IFNs and pro-inflammatory cytokines [96,114,115,116]. A decreased expression of PRRs implies a weaker innate antiviral response. Loske et al. reported that adults, who exhibit higher rates of SARS-CoV-2 infection and an increased risk of severe COVID-19 compared to children, demonstrated lower levels of PRRs, such as MDA5 and RIG-I, in the upper airway epithelial cells and innate immune cells compared to children [117].

SARS-CoV-2 infection is characterized by a significant lack of IFN production and secretion [118,119]. A notable difference between SARS-CoV-2 and influenza A virus infection is the poor induction of type I IFN response in COVID-19 [120,121]. Hadjadj et al. reported that patients with severe COVID-19 demonstrated a low or no IFN-α response and an absence of circulating IFN-β [121]. However, the mechanisms underlying these delayed and inefficient type I IFN responses in SARS-CoV-2 infection remain unclear. Several SARS-CoV-2 proteins have recently been reported as antagonists of the type 1 IFN response. NSPs, structural proteins M and N, and accessory proteins (ORF3a, 3b, 6, 7a, and 9b) interfere with type I IFN signaling directly or indirectly [122,123,124,125]. Some of them also degrade factors related to the IFN pathway via autophagy. ORF9b antagonizes type I and III IFN by targeting multiple signaling pathways, including RIG-I/MDA-5-MAVS, TLR3-TRIF, and cGAS–STING [126]. NSP15 suppresses type I IFN production by inhibiting phosphorylation and the nuclear translocation of IRF3 [127]. NSP13 inhibits type I IFN production by degrading TBK1 via p62-dependent selective autophagy [128].

pDCs act as the primary source of type I IFN upon detecting SARS-CoV-2-infected cells in the nasal mucosa. The sensing mechanism of SARS-CoV-2-infected cells by pDCs requires integrin-mediated cell adhesion (αLβ2 integrin and ICAM-1), allowing them to efficiently inhibit viral replication through a local response at the contact site with infected cells [129]. According to recent studies, the loss of pDC response was observed in severe COVID-19 [129,130].

Following poor IFN I and III responses, patients with severe COVID-19 fail to suppress viral replication in the early phase of the infection, leading to an exaggerated inflammatory response in the late phase. The dysregulated release of pro-inflammatory cytokines may contribute to life-threatening immune responses in COVID-19, such as a cytokine storm mediated by inflammatory cell death (PANoptosis). PANoptosis is primarily induced by the synergism of TNF-α and IFN-γ and is dependent on the activation of the JAK/STAT1/IRF1 axis and the subsequent activation of caspase-8 to drive cell death [131].

Analyses of human airway specimens have supported the essential role of the local innate immune response. A single-cell RNA sequencing analysis of nasopharyngeal swab samples from patients with COVID-19 showed that epithelial cells from severe cases exhibited the blunted expression of IFN-responsive or antiviral genes, suggesting that impaired antiviral immune responses in the nasal epithelium may underlie severe disease [132]. Additionally, IFN-α2 levels in endotracheal aspirate from patients with COVID-19 negatively correlated with the duration of hospital stay [133]. In contrast, an exaggerated innate immune response and the augmented recruitment of immune cells may contribute to tissue injury in patients with severe COVID-19. Patients with critical COVID-19 have been shown to exhibit stronger interactions between epithelial and immune cells and a higher activation status of inflammatory macrophages expressing *CCL2*, *CCL3*, *CCL20*, *CXCL1*, *CXCL3*, *CXCL10*, *IL8*, *IL1B*, and *TNF* than moderate cases [4].

Of note, the innate cellular response in the airways of pediatric patients differs from that observed in adults. In the nasal airways, the local innate IFN response to SARS-CoV-2 is stronger in pediatric immune cells compared with adult immune cells. A previous study reported that the airway epithelium showed a higher steady-state expression of IFN-response genes in children [134]. Pre-stimulation with IFNs may restrict viral spread and underlie mild diseases in children. Many types of innate immune cells also had elevated IFN response signatures in children compared to adults, particularly CD56^lo^ natural killer cells, natural killer T cells, neutrophils, and CXCL10^+^ monocytes [134].

## 5. Adaptive Immunity to SARS-CoV-2 Infection in the Airway

### 5.1. T-Cell Responses against SARS-CoV-2 in the Airway

**Figure 1:**
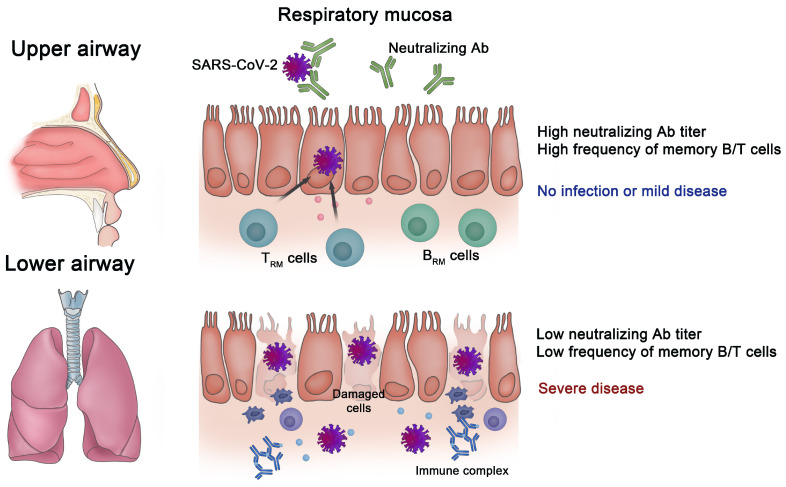
Importance of immune memory in the respiratory mucosa for protection against SARS-CoV-2. When there are high titers of neutralizing antibodies (NAbs) and a substantial number of tissue-resident memory T (T_RM_) and tissue-resident memory B (B_RM_) cells in the mucosa of the respiratory tract, infection with severe acute respiratory syndrome coronavirus 2 (SARS-CoV-2) is blocked, or it only results in mild disease. However, the progression to severe disease occurs when there are low titers of NAbs and weak memory B/T cell responses in the respiratory mucosa.

As with infections with other respiratory viruses, T_RM_ cells have been suggested to play a critical role in rapid protection against SARS-CoV-2 infection (Figure 1). After the resolution of the natural infection, SARS-CoV-2-specific T cells have been found in various tissues, including the bone marrow, spleen, lungs, and lymph nodes [135]. These SARS-CoV-2-specific T cells persist for at least six months after infection, and their frequency correlates with that of circulating T cells. In addition, a previous T-cell receptor sequencing analysis showed that CD8^+^ T cells carrying SARS-CoV-2-specific T-cell receptors were observed in nasal tissue after the resolution of SARS-CoV-2 infection [136]. However, the longevity of SARS-CoV-2-specific airway T_RM_ cells following natural infection remains enigmatic.

Intriguingly, previous studies using stimulation-based functional assays have shown that SARS-CoV-2-reactive T cells expressing canonical T_RM_ markers, including CD69 and CD103, are detected in the tonsils and BALF samples of individuals unexposed to SARS-CoV-2 [137,138]. These SARS-CoV-2-reactive T cells were thought to be induced by previous infection with other common cold coronaviruses. Although these tissue-resident cross-reactive T cells were shown to recognize multiple SARS-CoV-2 epitopes present in structural and nonstructural proteins, the protective role of these cells needs to be addressed.

Mucosal-associated invariant T (MAIT) cells are a subset of innate T cells that are involved in mucosal immunity and protection against viral infections. Several studies showed reduced frequencies and activated phenotypes of circulating MAIT cells in patients with COVID-19. In contrast, a significant enrichment of MAIT cells with activated phenotypes was observed in the airways, suggesting a potential contribution to the regulation of local infection and inflammation in patients with COVID-19 [139]. Additionally, others suggested that altered MAIT cell functions due to IFN-α–IL-18 imbalance may contribute to the disease severity of COVID-19 [140]. Remarkably, the frequency of circulating MAIT cells was restored in convalescent patients, indicating dynamic recruitment to the tissues during the acute phase and subsequent release back into the circulation after the resolution of the disease [141]. These findings collectively indicate that MAIT cells are involved in the immune response against SARS-CoV-2 and suggest their possible contribution to COVID-19 immuno-pathogenesis.

Since the administration of COVID-19 vaccines, whether intramuscular vaccination induces SARS-CoV-2 S-specific T_RM_ cells in the respiratory tract has been a topic of scientific interest. Several studies have performed stimulation-based assays to investigate the presence of SARS-CoV-2-reactive T cells in airway samples of vaccinees. One study identified S-specific T cells in nasopharyngeal swab samples from vaccinees [142], consistent with recent findings that mRNA vaccination induces T_RM_ cell generation in a mouse model [143]. Another study also reported that S-specific CD4^+^ T cells were detected in the lung tissue of vaccinated patients, although polyfunctional CD107a^+^IFN-γ^+^ T_RM_ cells were virtually absent in vaccinated individuals [144]. These results indicate that mRNA vaccination induces SARS-CoV-2 S-specific T cell responses in the lungs, although to a limited extent. In contrast, a study analyzing paired PB and BALF samples from vaccinated individuals without breakthrough infections showed that the frequency of S-reactive T cells was significantly lower in BALF than in PB [145]. In another study, SARS-CoV-2-reactive T cells were not detected in the nasal secretions of vaccinees without infection [146]. Furthermore, a recent analysis of BALF samples reported that vaccination alone did not elicit S-specific T cell responses that were significantly greater than those in pre-pandemic samples [147].

Apart from the controversy surrounding whether intramuscular vaccination induces SARS-CoV-2 S-specific T_RM_ cells in the airways, a growing body of evidence from animal models has demonstrated the superior ability of intranasal vaccination to induce airway T_RM_ cells [143,148,149,150]. Therefore, innovative vaccines with different routes of administration are being developed, with a focus on inducing greater mucosal immunity that can provide durable protection at the site of infection.

### 5.2. Humoral Immune Responses to SARS-CoV-2 in the Airway

Although circulating antibodies in PB contribute to viral clearance, the successful generation of mucosal memory B cells allows for a rapid increase in the local antibody titer, which could mediate efficient viral clearance and the prevention of viral spread upon reinfection (Figure 1) [151]. As described above, memory B cells have been identified in multiple mucosal tissues, including the lungs of mice, following influenza virus infection [76]. Similarly, a previous study showed that following a natural infection, SARS-CoV-2-specific memory B cells were induced in multiple human tissues, including the bone marrow, spleen, lungs, and lymph nodes [135]. Furthermore, IgA is a key component of the mucosal immune response to SARS-CoV-2. Virus-specific IgA antibodies are produced by antibody-secreting cells in the mucosal-associated lymphoid tissues, such as the palatine tonsils, and are secreted into the respiratory tract, where they play several important roles in mucosal defense, such as preventing the entry of the virus and reducing transmission. SARS-CoV-2-specific IgA antibodies were detected in the saliva and BALF of patients with COVID-19, and SARS-CoV-2 neutralization was more closely correlated with IgA than with IgM or IgG in the first weeks after symptom onset [152]. Recent studies also showed that wild-type SARS-CoV-2 spike-specific nasal IgA antibodies are associated with protection against infections with SARS-CoV-2 variants, including Omicron BA.1, BA.2, and BA.5 [153,154]. However, another study reported waning nasal SARS-CoV-2-specific IgA antibodies nine months after COVID-19 [155], and these antibodies were not induced by intramuscular COVID-19 vaccination [156]. These data raise the need to develop novel vaccine strategies.

Interestingly, adults and children exhibit different IgA mucosal antibody responses to SARS-CoV-2. Some studies, particularly in children, have reported that mild or low antigen exposure might promote mucosal SARS-CoV-2-specific IgA responses, showing an earlier IgA mucosal immune response to SARS-CoV-2 [157,158]. The levels of SARS-CoV-2 S protein-specific IgA in nasal fluid inversely correlated with age [157]. In addition, a longitudinal study by Chan et al. reported that early and robust nasal S1-specific IgA levels are linked to a rapid decline in viral load, as evidenced by analyses of SARS-CoV-2 S1-specific IgA levels and viral titers in nasal epithelial lining fluid [158]. In the study, pediatric patients, especially those who were asymptomatic, exhibited a rapid induction of IgA within the initial four days post diagnosis, while a noteworthy increase in IgA was detected only between 12 and 16 days post diagnosis in adult patients [158]. These findings may also support why children showed a lower risk of SARS-CoV-2 infection and milder disease status.

The palatine tonsils and adenoids are secondary lymphoid structures that are located at the nasopharynx and oropharynx, where antigen-specific T and B cell responses in the upper respiratory tract can be generated. It was reported that tonsils and adenoids are sites of the persistence of SARS-CoV-2 in children even without symptoms [159]. Therefore, memory B cells in the palatine tonsils and adenoids may play a pivotal role in immune defense against SARS-CoV-2. A recent study analyzing tonsil and adenoid tissues from pediatric patients with COVID-19 showed that the majority of SARS-CoV-2 S1-specific B cells from tonsils and adenoids exhibited distinct phenotypes, characterized by a higher expression of CXCR3 and HOPX and a lower expression of several inhibitory receptors, including FCGR2B, FCRL2, FCRL3, and TNFRSF13B [160]. In BCR sequencing analyses, S1^+^ B cells were primarily IgG1 and IgA1 class-switched cells, with high frequencies of somatic hypermutation (SHM) in VH genes and a low clonal diversity compared to S1− B cells, indicating their antigen-driven clonal expansion and GC origin [160]. In that study, the frequencies of S1^+^RBD^+^ B cells in adenoids significantly correlated with serum neutralization titers for B.1.351 (Beta), B.1.526 (Iota), B.1.617.2 (Delta), and B.1.1.529 (Omicron) variants [160], suggesting a critical role for adenoid B cells in generating immune responses to SARS-CoV-2. Furthermore, GC B cells are expanded in adenoids after COVID-19. These results provide evidence for persistent SARS-CoV-2-specific B cell responses in pharyngeal lymphoid tissues following infection.

Tertiary lymphoid structures (TLSs) often develop at sites of inflammation and are key sites in which memory B cells are reactivated, giving rise to antibodies that are capable of mediating rapid viral clearance. However, it remains unclear whether virus-specific B_RM_ cells arise from germinal center responses in TLSs, such as inducible bronchus-associated lymphoid tissue, located in mucosal tissues, or whether they migrate to the mucosal tissue after development in the draining lymph node. Additional studies are required to elucidate the presence and role of TLSs in the airways regarding humoral immune responses to SARS-CoV-2 and other respiratory viruses.

Similar to mucosal cellular immunity, the development of novel vaccination approaches that induce mucosal memory B cell responses is required. Studies have shown that an intranasal adenovirus-based COVID-19 vaccine induced mucosal B cells and antibodies in mice [161] and rhesus macaques [162] and was protective against upper and lower airway infections. In a hamster model, the intranasal delivery of an adenovirus-based vaccine generated a robust neutralizing antibody response and provided better protection than intramuscular delivery [163]. Additionally, Diallo et al. demonstrated that an intranasally delivered S protein trimer with adjuvant potently elicited S-specific IgG and IgA antibodies in the nasal cavity and lungs [150]. Collectively, these results indicate that vaccines capable of eliciting humoral responses in mucosal tissues may be effective strategies for inducing protective immunity.

## 6. Alterations in Mucosal Immune Responses against SARS-CoV-2 in the Allergic Airway Diseases

### 6.1. Differential Expression of Viral Entry-Related Receptors in the Allergic Airway

Factors involved in viral entry into respiratory epithelial cells can be influenced by environmental stimuli within the airway. Distinct expression patterns of these entry-related genes have been reported in allergic airway inflammation, primarily mediated by type 2 cytokines such as IL-4, 5, and 13. Several studies of SARS-CoV-2 have indicated a significant decrease in ACE2 expression and an increase in TMPRSS2 expression in airway epithelial cells in response to type 2 inflammation [164,165,166,167,168,169]. Kimura et al. found that IL-13 significantly reduced ACE2 expression and increased TMPRSS2 expression in primary BECs. Similar expression patterns for ACE2 and TMPRSS2 were observed in nasal epithelial cells and BECs in type 2 asthma and AR, which were mainly mediated by type 2 inflammation [167]. Coden et al. also reported that ACE2 was downregulated and TMPRSS2 was upregulated in BECs from patients with Th2-high asthma compared with those with Th2-low asthma [168]. In addition, pre-stimulation with IL-13 before SARS-CoV-2 infection led to a reduction in viral replication and ACE2 protein levels in BECs from children with allergic asthma compared with those from control subjects [170]. This finding suggests a protective effect of type 2 inflammation against SARS-CoV-2 entry. Consistent with these results, a previous study analyzing 900 patients infected with SARS-CoV-2 showed that patients with type 2 inflammation had lower viral loads upon entry into the study [171]. Conversely, several studies have reported that patients with COVID-19 have a lower prevalence of asthma and AR than the general population [172,173,174].

In contrast, ACE2 expression in airway epithelial cells increases in the presence of type 1 inflammation or a low Th2 profile. Saheb et al. found that an increase in IL-17, a cytokine associated with type 2-low asthma, was significantly associated with a higher ACE2 expression in BECs [175]. In an in vitro study using airway epithelial cells, Ziegler et al. discovered that ACE2 is a human IFN-stimulated gene (ISG), demonstrating that it was elevated in response to type I IFNs and, to a lesser extent, type II IFNs [166].

Collectively, these results show that the expression of SARS-CoV-2 entry-related receptors may undergo distinctive changes in the allergic airway.

### 6.2. Innate and Adaptive Immune Responses

In allergic airways, impaired innate immune responses to viral infections have been reported; these are associated with a biased immune response towards Th2 polarity and are primarily mediated by type 2 inflammation [176]. Type 2 inflammation is characterized by elevated eosinophils in the airways or PB and an abundance of type 2 cytokines. Gilles et al. reported that the type 2 cytokines IL-4 and IL-13 could impair the immune response to rhinovirus 16 infection by inhibiting TLR3 expression and IRF3 signaling [177]. Additionally, allergic inflammation and eosinophilia induced by IL-5 suppresses TLR7 expression in the lungs [178]. Eosinophils may suppress TLR7 responses and weaken antiviral responses; however, further investigations are required to understand the impact of TLR7 downregulation on SARS-CoV-2 infection.

The innate immune response can be modulated by allergens such as pollen and HDM. Akbarshahi et al. reported that in human BECs and a mouse model of asthma, exposure to HDM before viral infection decreased the IFN-β and IFN-λ response by affecting the TLR3 signaling pathway [179]. Pollen exposure can also affect the immune responses to viral infections. Hajighasemi et al. proposed that pollen exposure directly affects antiviral defense within the airway and enhances susceptibility to SARS-CoV-2 infection [180]. Another study reported that exposure to pollen during viral infections reduced the production of proinflammatory chemokines and type I/III IFNs, and increased viral replication [177]. Additionally, several proteases within the pollen may disrupt epithelial barrier integrity and function, similar to HDM, resulting in the increased permeability of the subepithelial layers by respiratory viruses [181].

Contrary to the previously described impaired immune response in allergic airways, some studies have suggested a protective role of allergic airway inflammation in antiviral defense. One study investigated the potential influence of AR on the immune response following vaccination with a two-dose inactivated SARS-CoV-2 vaccine. The results indicated that patients with pre-existing AR demonstrated a Tfh2 cell-associated enhanced humoral immune response to the inactivated SARS-CoV-2 vaccine compared with the control subjects. Furthermore, elevated levels of neutralizing antibodies were observed at 10–12 months post infection in the AR group compared to those in the control group [182]. Consistent with these results, Chen et al. investigated the levels of SARS-CoV-2-specific humoral and cellular immunity in patients with and without asthma, and found that the level of SARS-CoV-2-specific neutralizing antibodies was higher in COVID-19 survivors with asthma than in those without asthma at the eight-month follow-up, whereas no significant differences were noted in the cellular immunity levels between the two groups. This implies that patients with asthma may benefit from augmented humoral immunity during the recovery period from COVID-19 [183]. Additionally, a positive correlation between the levels of SARS-CoV-2-specific T cell memory responses and the blood eosinophil and regulatory T cell percentages was observed, suggesting a potential protective role of eosinophils in antiviral host defense [183]. Ferastraoaru et al. found that patients with SARS-CoV-2 infection and pre-existing eosinophilia (absolute eosinophil count ≥ 150 cells/μL) also showed a decreased likelihood of hospitalization and mortality [184]. Similarly, a retrospective cohort study on patients with SARS-CoV-2 infection reported that patients with a blood eosinophil count greater than or equal to 200 cells/μL demonstrated lower mortality regardless of the presence of asthma [185].

### 6.3. Susceptibility to Infection and Clinical Outcomes of COVID-19

The effect of allergic airway inflammation on susceptibility to SARS-CoV-2 infection and clinical outcomes is complicated and controversial. A study of 70,557 patients who underwent SARS-CoV-2 testing found that AR was associated with a lower risk of SARS-CoV-2 infection. Asthma exhibited a similar protective effect against SARS-CoV-2 infection in patients aged under 65 years, despite its higher risk of hospitalization than that in healthy controls [186]. Furthermore, a large retrospective cohort study of patients with SARS-CoV-2-induced pneumonia found a significant association between atopic status and mild COVID-19 [187]. In contrast, in a nationwide Korean cohort, asthma and AR were associated with an increased likelihood of SARS-CoV-2 infection and worse clinical outcomes; however, patients with non-allergic asthma had a greater risk of SARS-CoV-2 test positivity and more severe clinical outcomes than those with allergic asthma [188]. Similarly, in a propensity-score-matched nationwide cohort study, patients with AR had a higher risk of developing SARS-CoV-2 infection [189]. Certainly, it is necessary to consider the severity of comorbid diseases. A national cohort study in the UK reported an increased risk of hospitalization owing to COVID-19 among patients with severe or poorly controlled asthma compared to those without asthma. However, patients with mild or well-controlled asthma did not show a significantly increased risk of hospitalization and mortality from COVID-19 compared with those without asthma [190].

## 7. Concluding Remarks and Perspectives

An extensive body of research conducted during the COVID-19 pandemic has substantially advanced our understanding of immune responses against SARS-CoV-2 and respiratory viruses. However, most studies have relied on easily accessible PB. Currently, there is a growing need for more detailed investigations of the features and protective roles of mucosal immune responses. Mounting evidence from animal studies has shown that the establishment of neutralizing antibodies and immune memory in the respiratory tract leads to superior protection against invading respiratory viruses. Table 1 summarizes the key components of mucosal immune responses against SARS-CoV-2. However, the regulatory mechanisms governing the generation and maintenance of immune memory in the respiratory tract are not fully understood. In addition, the exact impact of respiratory diseases, such as allergic airway inflammation, on mucosal antiviral defense needs to be further addressed. Further mechanistic investigations coupled with data from human cohort studies will provide novel insights into the development of effective vaccines that induce greater mucosal immunity.

## Figures and Tables

**Table 1:** A summary of the key components of mucosal immune responses against SARS-CoV-2.

Immunity	Component	Roles and/or Findings	Refs.
Innate immunity	Mucins (soluble and transmembrane mucins)	Controversial roles: elevated levels of MUC1 and MUC5AC (soluble mucins) may impact disease progression, while transmembrane mucins show in vitro evidence of protective functions by preventing SARS-CoV-2 binding and entry.	[103,104,105,106,107]
	Antimicrobial peptides (AMPs)	Increased β-defensin transcripts are observed in SARS-CoV-2 patients; potential role in inhibiting viral entry.	[108,109]
	Type I IFN response	Delayed and inefficient induction in COVID-19 contributes to severe disease. Inborn defects or autoantibodies against type I IFNs; higher risk of severe COVID-19.	[96,101,102,113,114,115,116,117,118,119,120,121,122,123,124,125,126,127,128]
	Plasmacytoid DCs (pDCs)	Primary source of type I IFNs; loss of response observed in severe COVID-19.	[129,130]
	Cytokine storm (PANoptosis)	Life-threatening immune response mediated by inflammatory cell death; the synergism of TNF-α and IFN-γ.	[131]
Adaptive immunity	Tissue-resident memory (T_RM_) cells	Critical role in rapid protection; longevity remains unclear. Increasing in vivo evidence supporting superior ability of intranasal vaccination to induce airway T_RM_ cells.	[135,136,146]
	Mucosal-associated invariant T (MAIT) cells	Enrichment of MAIT cells and activated phenotypes in the airway of patients with COVID-19; potential role in local immune response.	[139,140,141]
	Mucosal memory B cells	Rapid increase in local antibody titer for efficient viral clearance. SARS-CoV-2-specific B cells in tonsils and adenoids exhibit distinct phenotypes and play potential pivotal role in immune defense, especially in pediatric patients.	[135,151,159,160]
	IgA	Key component of mucosal immune response; more closely correlated with SARS-CoV-2 neutralization than IgM or IgG. Children exhibited earlier and more robust mucosal IgA response to SARS-CoV-2; linked to a rapid decline in viral load.	[152,153,154,157,158]

## Data Availability

No new data were created or analyzed in this study. Data sharing is not applicable to this article.
